# Incorporating parenchymal heterogeneity into FLIS to improve MRI-based liver function assessment

**DOI:** 10.1186/s13244-025-02187-z

**Published:** 2026-01-12

**Authors:** Hande Özen Atalay, Muhammet Selman Sogut, Murat Akyildiz, Afak Durur Karakaya

**Affiliations:** 1 Radiology Department, Koc University Hospital, Istanbul, Turkey; 2 Anesthesiology and Reanimation Department, Koc University Hospital, Istanbul, Turkey; 3 Gastroenterology and Hepatology Department, Koc University Hospital, Istanbul, Turkey

**Keywords:** Magnetic resonance imaging, Liver function, Elastography, Liver cirrhosis, Functional liver imaging score

## Abstract

**Objectives:**

To assess the correlation between the functional liver imaging score (FLIS) and FibroScan^®^-derived fibrosis stage, and to determine whether incorporating parenchymal heterogeneity (FLIS-H) improves its association with fibrosis and clinical scores.

**Materials and methods:**

This retrospective single-centre study included 113 patients who underwent FibroScan^®^ and hepatocyte-specific contrast-enhanced MRI within a median interval of 4 days. FLIS was calculated, and the parenchymal heterogeneity score was added to FLIS (FLIS-H; range 0–8). Inter-reader agreement was evaluated using a two-way random-effects intraclass correlation coefficient (ICC). Correlations between FLIS/FLIS-H and fibrosis stage/clinical scores (Child–Pugh, MELD, ALBI) were assessed using Spearman’s rank correlation. Steiger’s *z*-test and Zou’s method were used to compare correlations.

**Results:**

A total of 113 patients (67 men; mean age 56.6 ± 13.5 years) were evaluated. Inter-reader agreement was excellent for FLIS (ICC 0.994; 95% CI: 0.975–1.000), heterogeneity (ICC 0.949; 95% CI: 0.901–0.984), and FLIS-H (ICC 0.974; 95% CI: 0.957–0.989). FLIS showed significant negative correlations with Child–Pugh (ρ = −0.2664, *p* = 0.0087), ALBI (ρ = −0.3076, *p* = 0.0022), and fibrosis stage (ρ = −0.3207, *p* < 0.001). FLIS-H demonstrated stronger correlations with Child–Pugh (ρ = −0.4167, *p* < 0.001), ALBI (ρ = −0.5243, *p* < 0.001), MELD (ρ = −0.2360, *p* = 0.020), and fibrosis stage (ρ = −0.5270, *p* < 0.001). Steiger’s *z*-test confirmed that correlations were significantly improved with FLIS-H for ALBI (*z* = −3.03, *p* = 0.0025), Child–Pugh (*z* = −2.01, *p* = 0.045), and fibrosis stage (*z* = −2.90, *p* = 0.0038).

**Conclusion:**

FLIS correlates significantly with fibrosis stage and clinical scores. Incorporating parenchymal heterogeneity into FLIS enhances these associations and may provide a superior method for liver assessment.

**Critical relevance:**

This study introduces a modified FLIS version (FLIS-H) that integrates parenchymal heterogeneity and demonstrates superior correlation with elastography-derived fibrosis stages and clinical scoring systems, offering a practical improvement for non-invasive assessment in routine practice.

**Key Points:**

- FLIS has no reported correlation with elastography-based liver fibrosis staging.
- Parenchymal heterogeneity is not included as a parameter in the standard FLIS.
- Integrating heterogeneity improves correlation with fibrosis stage and clinical scores.
- FLIS-H enables fast, reliable, structure-function liver assessment in clinical radiology.

**Graphical Abstract:**

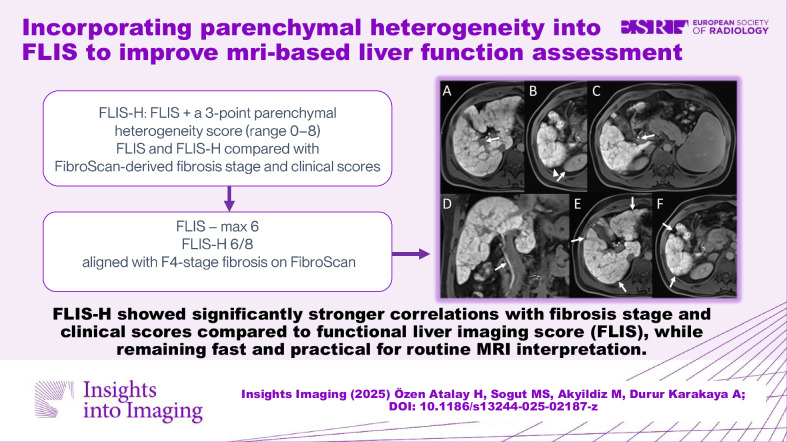

## Introduction

Chronic liver disease (CLD) represents a steadily rising global health burden, accounting for almost two million deaths annually [1]. The aetiology of CLD shows geographic variation, with viral hepatitis more common in Asia and sub-Saharan Africa, and alcohol-related liver disease (ALD) and metabolic dysfunction-associated steatotic liver disease (MASLD) more frequent in Western countries [2, 3]. Regardless of cause, progressive hepatocellular injury and fibrosis remain the common pathway leading to functional decline [4, 5].

Although liver biopsy remains the reference standard, its invasiveness, small sample size, sampling variability and potential complications have driven a shift toward non-invasive methods in liver assessment [6, 7]. Transient elastography (FibroScan^®^) provides rapid quantification of liver stiffness and steatosis, but performance can be limited by body habitus, inflammation and operator dependency [8]. Gadoxetic acid-enhanced MRI offers whole-organ morphological and hepatocyte-specific functional evaluation, enabling qualitative assessment of liver parenchyma [9, 10].

Within this context, the functional liver imaging score (FLIS) was introduced in 2016 for orthotopic liver transplantation and adapted to native livers in 2018 [11, 12]. FLIS sums three qualitative hepatobiliary-phase features—parenchymal enhancement, biliary contrast excretion and portal vein signal intensity—yielding a 0–6 scale in which lower scores reflect impaired function. Subsequent studies demonstrated significant correlations between FLIS and clinical metrics such as Child–Pugh, model for end-stage liver disease (MELD) and albumin-bilirubin (ALBI) scores, as well as its ability to predict decompensation, post-hepatectomy liver failure and cancer-related cachexia [13–17].

Nevertheless, two important gaps remain. First, the relationship between FLIS and quantitative fibrosis measurements derived from FibroScan^®^ has not yet been examined, although fibrosis is a principal driver of functional decline. Second, FLIS does not account for macroscopic parenchymal heterogeneity—a hallmark of advanced fibrosis that becomes increasingly conspicuous on hepatobiliary-phase MRI [18]. Anecdotally, during routine practice, we encountered patients with identical FLIS values but markedly different heterogeneous enhancement patterns, raising the question of whether integrating heterogeneity could enhance prognostic performance.

The present study addresses these gaps through a retrospective analysis of patients who underwent both FibroScan^®^ and gadoxetic acid-enhanced MRI within a 90-day interval. The primary objective of this study was to evaluate the association of FLIS and a modified score, functional liver imaging score with parenchymal heterogeneity (FLIS-H), which incorporates a simple three-point assessment of parenchymal heterogeneity into FLIS with elastography-derived fibrosis stage and established clinical liver scores (Child–Pugh, MELD, and ALBI). By clarifying the fibrotic and functional relevance of FLIS and FLIS-H, our goal is to facilitate broader, more reliable application of non-invasive MRI biomarkers in routine liver evaluation.

## Materials and methods

This retrospective observational study was approved by the Institutional Research Ethics Committee of our hospital (Institutional Review Board approval number: 2024.393.IRB2.175) and conducted in accordance with the principles of the Declaration of Helsinki. Owing to its retrospective design, the requirement for written informed consent was not required.

### Study population

All adult patients (≥ 18 years) who had undergone both (i) FibroScan^®^ assessment in the gastroenterology department and (ii) a dynamic upper-abdominal MRI examination with a hepatocyte-specific contrast agent between February 2022 and September 2024 were included in the study. Imaging was performed for various indications related to suspected or established CLD. Exclusion criteria were the presence of congestive hepatopathy, acute hepatitis, chronic portal vein thrombosis, and diffuse metastatic liver disease, since these conditions might confound the planned correlations between clinical scores, FibroScan^®^ measurements and FLIS evaluation. Additionally, patients with an interval between two examinations > 90 days were excluded. For patients with more than one MRI–FibroScan^®^ pair, the temporally closest pair was selected for analysis.

### FibroScan^®^ protocol and clinical data collection

All FibroScan^®^ examinations were performed by a gastroenterologist (M.A.) with 20 years of elastography experience. After the patient had fasted for at least 4 h, the examination was conducted with the M-probe or XL-probe, as dictated by the patient’s body mass index, and measurements were obtained from the right hepatic lobe according to routine clinical practice. Ten valid measurements were required, and studies with an inter-quartile-range-to-median ratio (IQR/M) > 30% were rejected. Fibrosis was staged based on liver stiffness (E, kPa) with aetiology-specific cut-offs derived from published studies [19–27].

To compute clinical scores, all laboratory data and relevant clinical information were extracted from the electronic medical record: serum albumin (g L⁻¹), international normalized ratio (INR), total bilirubin (mg dL⁻¹), serum creatinine (mg dL⁻¹), platelet count, aspartate aminotransferase, alanine aminotransferase, gamma-glutamyl transferase and alkaline phosphatase. The presence and grade (minimal or moderate) of ascites were recorded from either contemporaneous imaging or clinical notes. Hepatic encephalopathy was determined from the same clinical encounter. All laboratory values used for score calculation were obtained on the same day as the FibroScan^®^ examination, in accordance with routine clinical workflow. Scores were calculated as follows:

- MELD = 9.57 × ln(creatinine) + 3.78 × ln(bilirubin) + 11.20 × ln(INR) + 6.43 [28].
- ALBI = (log₁₀ bilirubin [µmol L⁻¹] × 0.66) + (albumin [g L⁻¹] × −0.085) [29].
- Child–Pugh was derived from total bilirubin, albumin, INR, ascites and encephalopathy; patients were classified as Child–Pugh A (5–6 points), B (7–9 points) or C (10–15 points) [30]

### MRI examination protocol and radiologic image interpretation

Dynamic upper-abdominal MRI was performed on 1.5-T and 3-T systems (Magnetom Aera and Skyra, Siemens Healthineers) using the department’s routine protocol, which also included diffusion-weighted imaging. The protocol parameters are detailed in Table 1. The hepatocyte-specific contrast agent gadoxetic acid (Gd-EOB-DTPA, *Primovist®, Bayer*) was administered intravenously at 0.025 mmol/kg. Routine hepatobiliary-phase imaging was obtained at a minimum of 20 min.

**Table 1:** Upper abdominal MRI protocol acquired using Gd-EOB-DTPA

	TR/TE (ms)	Sequence	Flip angle	FOV (mm)	Slice thickness (mm)	Matrix size
Coronal T2W fat-saturated	1000/104	TSE	150	400 × 400	4	307 × 384
Axial T2W	1000/96	TSE	150	332 × 380	5	272 × 384
Axial T2W fat-saturated	1000/96	TSE	150	315 × 360	5	272 × 384
Axial in-phase	110/2,46	DIXON	70	315 × 360	5	448 × 512
Axial out-of-phase	110/1,23	DIXON	70	315 × 360	5	448 × 512
Axial pre-contrast T1W	358/1,3	3D VIBE	10	321 × 380	3	189 × 320
Axial post-contrast T1W—arterial	358/1,3	3D VIBE	10	321 × 380	3	189 × 320
Axial post-contrast T1W—portal	358/1,3	3D VIBE	10	321 × 380	3	189 × 320
Axial post-contrast T1W—venous	358/1,3	3D VIBE	10	321 × 380	3	189 × 320
Coronal post-contrast T1W—late phase	356 × 1,3	3D VIBE	10	380 × 380	3	240 × 320
Axial post-contrast T1W—late phase	358/1,3	3D VIBE	10	321 × 380	3	189 × 320
Axial post-contrast T1W—hepatobiliary phase	358/1,3	3D VIBE	10	321 × 380	3	189 × 320
Coronal post-contrast T1W—hepatobiliary phase	356 × 1,3	3D VIBE	10	380 × 380	3	240 × 320
Axial DWI (*b* = 50,800)	7000/57	EPI	90	329 × 380	5	232 × 268

*T2W* T2 weighted, *T1W* T1 weighted, *DWI* diffusion weighted image, *TR* time to repeat, *TE* time to echo, *TSE* turbo-spin-echo, *VIBE* volumetric interpolated breath-hold examination, *EPI* echo-planar imaging

Images were reviewed by two radiologists with 20 (A.D.K.) and 5 years (H.Ö.A.) of experience. The presence of hepatomegaly (coronal mid-clavicular length > 160 mm) and steatosis (in-phase/opposed-phase gradient-echo images; signal loss on opposed-phase) was noted.

### FLIS

FLIS was calculated on the 20-min hepatobiliary-phase image according to the original description [11]. Parenchymal enhancement was scored by comparing hepatic signal with the right renal cortex: hypointense = 0, isointense = 1 and hyperintense = 2. Contrast excretion was graded by the most distal location of visible gadoxetic acid within the biliary tree: none = 0, peripheral intra-hepatic ducts or right/left hepatic duct only = 1 and common hepatic duct, common bile duct or duodenum = 2. Portal-vein signal intensity relative to the liver was assigned as hyperintense = 0, isointense = 1 or hypointense = 2 (Table 2). The three sub-scores were summed to obtain a total FLIS ranging from 0 to 6.

**Table 2:** Description and scoring criteria of the FLIS parameters

1. Liver parenchymal enhancement	Assessment of liver parenchymal signal intensity relative to the right renal cortex on HBP	Hypointense	0
		Isointense	1
		Hyperintense	2
2. Contrast excretion	Assessment of the biliary excretion of the contrast agent in HBP	No excretion	0
		Presence of contrast agent in the peripheral intra-hepatic bile ducts or in the right or left hepatic duct	1
		Visualisation of contrast agents in the common hepatic duct, common bile duct, or duodenum	2
3. Portal vein signal	Assessment of portal vein signal intensity relative to liver parenchyma signal on HBP	Hyperintense	0
		Isointense	1
		Hypointense	2

### Parenchymal heterogeneity score

**Fig. 1:**
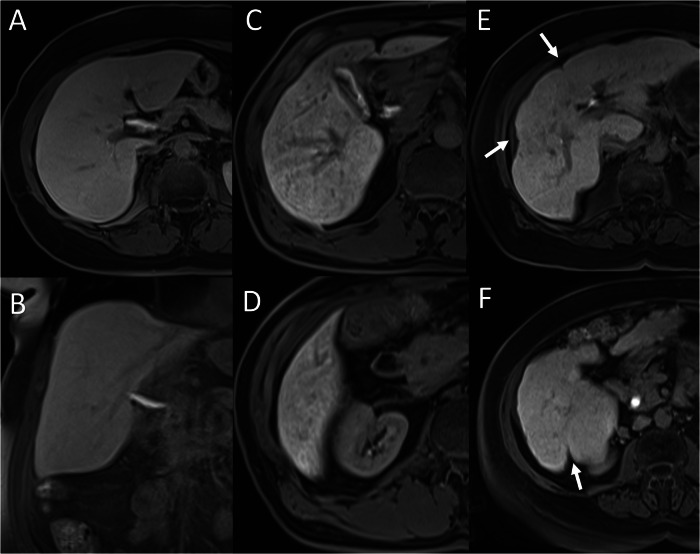
Examples of liver parenchymal heterogeneity scoring. **A**, **B** Homogeneous liver signal on hepatobiliary-phase images corresponds to a score of 2. **C**, **D** Heterogeneous parenchymal signal without capsular retraction corresponds to a score of 1. **E**, **F** Marked heterogeneity with associated capsular retraction (arrows) is scored as 0

On the same hepatobiliary-phase images, liver parenchyma was categorised as: homogeneous (score = 2), heterogeneous without capsular retraction (score = 1), or heterogeneous with capsular retraction (score = 0) (Fig. 1). The heterogeneity score was added to FLIS to create FLIS-H, ranging from 0 to 8.

### Statistical analysis

All analyses were performed in R software (version 4.4.1). Normality of continuous variables was assessed with the Shapiro–Wilk test. Because most variables were not normally distributed, Spearman’s rank correlation was used to examine associations between FLIS or FLIS-H and (i) fibrosis stage, (ii) Child–Pugh score, (iii) MELD score and (iv) ALBI score. Correlation coefficients (ρ) and corresponding *p* values are reported. Inter-reader reliability for FLIS, heterogeneity score, and FLIS-H was assessed using a two-way random-effects intraclass correlation coefficient (ICC) with 95 per cent confidence intervals.

To compare the strength of two dependent, overlapping correlations (e.g., FLIS vs FLIS-H with the same external variable), Steiger’s *z*-test was applied. Ninety-five per cent confidence intervals for correlation-difference estimates were calculated with Zou’s method. A two-sided *p* < 0.05 was considered statistically significant.

## Results

### Patient enrolment and baseline demographics

**Fig. 2:**
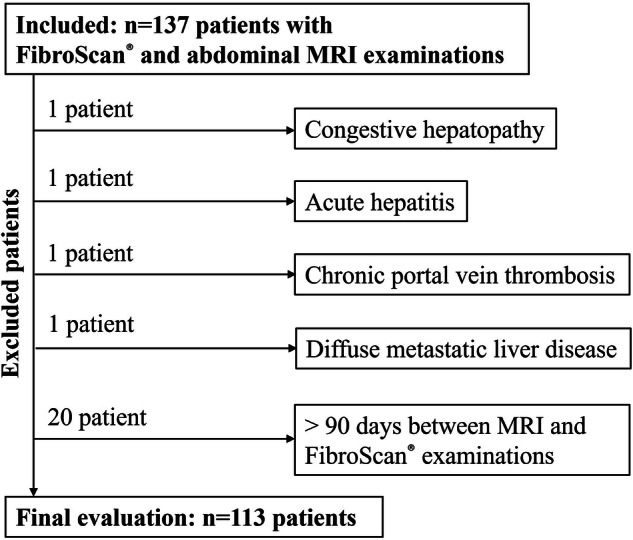
Flowchart of the study cohort

A total of 144 paired MRI/FibroScan^®^ exams were conducted on 137 patients. Seven patients had more than one MRI–FibroScan^®^ examination pair, and only the closest pair was included in the analysis. Following the exclusion of one patient with congestive hepatopathy, one with acute hepatitis, one with chronic portal vein thrombosis, one with widespread metastatic liver disease, and 20 patients with a study gap exceeding 90 days, the final sample comprised 113 unique patients (Fig. 2).

The group included 67 men (59.3%) and 46 women (40.7%) with a mean age of 56.6 ± 13.5 years. Aetiological analysis showed MASLD in 57 (50.4%), autoimmune hepatitis in 11 (9.7%), chronic hepatitis B in 11 (9.7%), ALD in 9 (7.9%), cryptogenic cirrhosis in 8 (7.1%), cholestatic disorders (primary biliary cholangitis, primary sclerosing cholangitis or IgG4-related sclerosing cholangitis) in 8 (7.1%), cholangiocarcinoma in 3 (2.7%), chronic hepatitis C in 3 (2.7%), and single cases of hereditary haemochromatosis, chemotherapy-associated liver injury and combined autoimmune hepatitis and primary sclerosing cholangitis (each 0.9%).

The median MRI—FibroScan^®^ interval was 4 days (range, 0–90). MRI identified steatosis in 80 patients (70.8%). Mean coronal long-axis liver length measured 166.9 ± 31.02 mm; hepatomegaly (> 160 mm) was present in 69/113 (61.1%).

The median Child–Pugh score was 5 (range 5–9), with 89 patients classified as class A and 7 as class B; no class C cases were observed. For MELD, the median value was 7 (range 6–18); 84 patients had scores below 10, whereas 13 scored 10 or higher. ALBI assessment yielded a median of −3.13 (range −4.00 to −1.45), corresponding to grade 1 in 84 cases and grade 2 in 13 cases. Notably, clinical scores could not be calculated in 17 patients for Child–Pugh (15.04%), 16 for MELD (14.16%), and 16 for ALBI (14.16%) due to missing laboratory data.

According to FibroScan^®^ measurements, median liver stiffness was 8.30 kPa (range 2.30–65.20): 54 (47.8%) F0-1, 13 (11.5%) F2, 11 (9.7%) F3 and 35 (31%) F4.

### FLIS, Parenchymal heterogeneity and FLIS-H

On hepatobiliary-phase images, parenchymal enhancement scored 0, 1 and 2 in 4 (3.5%), 20 (17.7%) and 89 (78.8%) patients, respectively. Biliary excretion scored 0 in 3 (2.7%), 1 in 1 (0.9%) and 2 in 109 (96.4%); portal-vein intensity scored 1 in 4 (3.5%) and 2 in 109 (96.4%). The composite FLIS distribution was: 1 (*n* = 2), 3 (*n* = 1), 4 (*n* = 4), 5 (*n* = 17), and 6 (*n* = 89).

Hepatobiliary-phase parenchyma was homogeneous (heterogeneity score = 2) in 69 (61.1%) patients, heterogeneous in 44 (38.9%) patients; of these, 23 showed no capsular retraction (score = 1), and 21 showed retraction (score = 0). Adding this parameter produced FLIS-H scores of 3 (*n* = 2), 5 (*n* = 9), 6 (*n* = 22), 7 (*n* = 22), and 8 (*n* = 58). Inter-reader agreement was excellent for FLIS (ICC 0.994; 95% CI: 0.975–1.000), parenchymal heterogeneity score (ICC 0.949; 95% CI: 0.901–0.984), and FLIS-H (ICC 0.974; 95% CI: 0.957–0.989).

### Correlation of FLIS, FLIS-H with clinical metrics and fibrosis stage

FLIS has a weak but statistically significant correlation with Child–Pugh score (ρ = −0.2664, *p* = 0.0087) and ALBI score (ρ = −0.3076, *p* = 0.0022). No significant relationship was observed with MELD (ρ = −0.1654, *p* = 0.106). A separate analysis demonstrated a weak but significant negative association between FLIS and elastography-derived fibrosis stage (ρ = −0.3207, *p* < 0.001) (Table 3).

**Table 3:** Correlation between clinical scoring systems, fibrosis stage and FLIS, FLIS-H

Functional and fibrosis parameters		FLIS	FLIS-H
Child–Pugh	Spearman correlation coefficient (ρ)	−0.2664	0.4167
	*p*-value	0.0087	< 0.001
MELD	Spearman correlation coefficient (ρ)	−0.1654	−0.2360
	*p*-value	0.1055	0.0199
ALBI	Spearman correlation coefficient (ρ)	−0.3076	−0.5243
	*p*-value	0.0022	< 0.001
FibroScan^®^-derived fibrosis stage	Spearman correlation coefficient (ρ)	−0.3207	−0.5270
	*p*-value	< 0.001	< 0.001

*FLIS* functional liver imaging score, *FLIS-H* functional liver imaging score with heterogeneity, *MELD* model for end-stage liver disease, *ALBI* Albumin–bilirubin

**Fig. 3:**
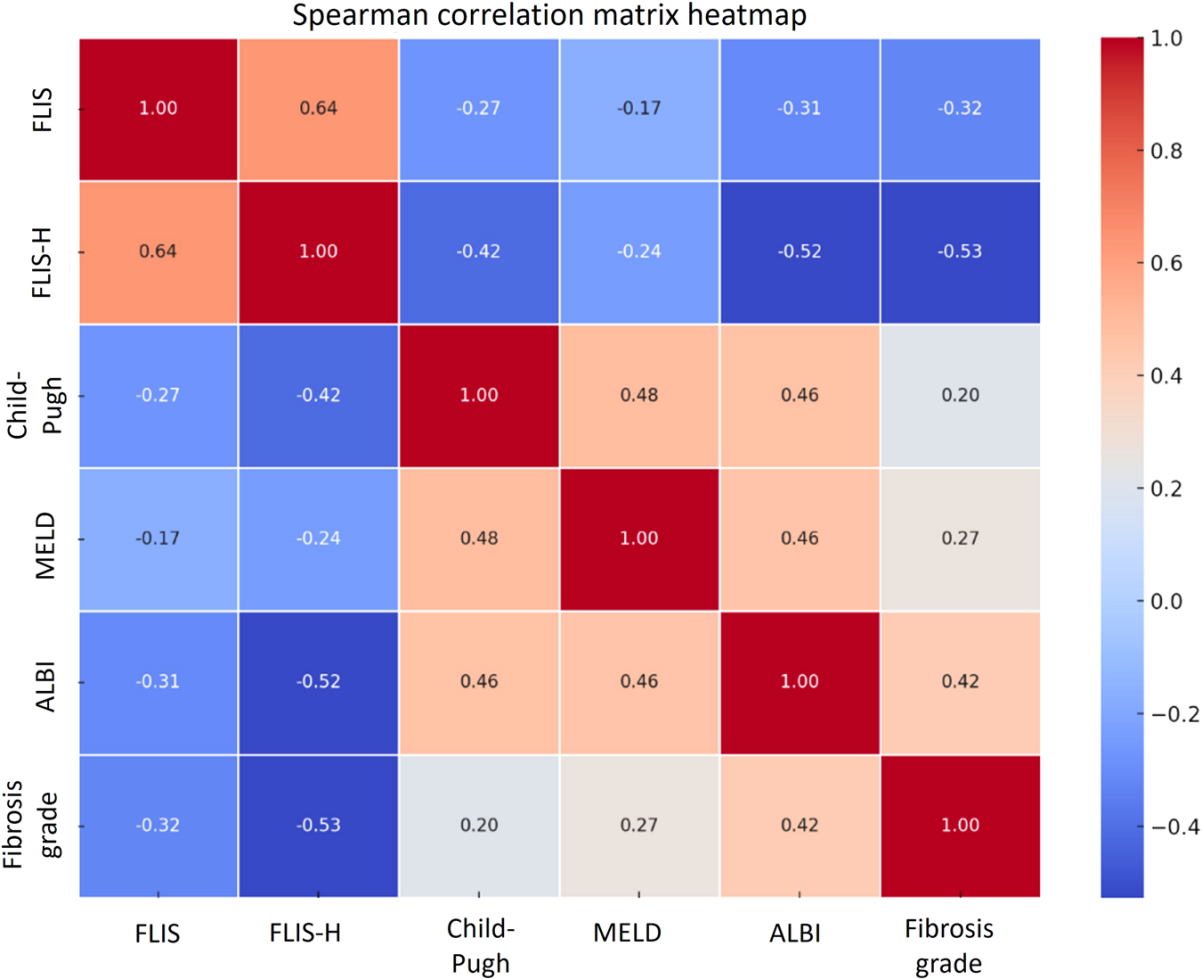
Correlation matrix of FLIS, FLIS-H, clinical parameters, and fibrosis stage

**Fig. 4:**
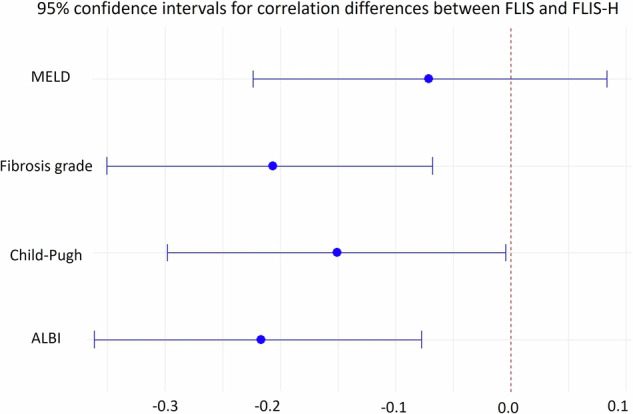
95% confidence intervals for correlation differences between FLIS and FLIS-H

Incorporating heterogeneity into FLIS (FLIS-H) increased all correlations with clinical scoring systems. FLIS-H showed moderate negative correlation with Child–Pugh (ρ = −0.4167, *p* < 0.001); ALBI (ρ = −0.5243, *p* < 0.001) and weak negative correlation with MELD (ρ = −0.2360, *p* = 0.02) (Table 3). Additionally, FLIS-H showed a moderate inverse correlation with fibrosis stage (ρ = −0.5270, *p* < 0.001) (Fig. 3). Steiger’s *z*-test and Zou’s 95% confidence interval confirmed that FLIS-H correlations were significantly stronger than FLIS for fibrosis stage (*z* = −2.90, *p* = 0.0038; 95% CI: −0.35 to −0.07), Child–Pugh (*z* = −2.01, *p* = 0.045; 95% CI: −0.30 to −0.00) and ALBI (*z* = −3.03, *p* = 0.0025; 95% CI: −0.36 to −0.08) (Fig. 4). The improvement over MELD did not reach significance (*z* = −0.89, *p* = 0.37).

## Discussion

In this study, we evaluated the correlations of FLIS with widely used clinical liver scores (Child–Pugh, MELD, and ALBI) and with fibrosis stage determined by transient elastography, using these clinical and elastographic measures as parallel reference benchmarks to assess the structural and functional relevance of MRI-based evaluation. In addition, we introduced a modified score, FLIS-H, obtained by adding a parenchymal-heterogeneity parameter to the original three FLIS components. Our analysis showed that FLIS already displays statistically significant weak inverse relationships with Child–Pugh, ALBI and elastography-based fibrosis stage. More importantly, FLIS-H yielded significantly stronger moderate correlations with the same variables, underscoring the complementary information provided by macroscopic heterogeneity.

Previous studies have firmly established FLIS as a convenient marker of global hepatocellular function [14, 17, 31]. Negligible inter-observer variability and near-perfect inter-rater agreement were confirmed in a meta-analysis of 1419 examinations, highlighting its importance in daily practice [32]. In line with this report, inter-reader agreement in our study was excellent for FLIS (ICC 0.994), as well as heterogeneity score (ICC 0.949), and the combined FLIS-H score (ICC 0.974), supporting the robustness of visual scoring in routine practice. However, to date, no study has examined whether FLIS reflects the mechanical stage of fibrosis assessed by transient elastography. Our finding of a weak but statistically significant negative correlation between FLIS and FibroScan^®^-derived fibrosis stage (ρ = −0.3207, *p* < 0.001) bridges this gap, and may suggest that reduced parenchymal enhancement, diminished biliary excretion and persistent portal-venous signal intensity represent imaging surrogates of the micro-architectural distortion that elevates liver stiffness.

Although FLIS has been shown to have moderate to strong correlations with Child–Pugh and ALBI grades (ρ = −0.538 to −0.854) in previous cohorts [14, 17, 31], the weak associations seen in our study (ρ = −0.2664 and −0.3076, respectively) likely reflect the heterogeneity of our patient population, which encompassed the full aetiological spectrum of CLD such as steatotic, viral, alcoholic, cholestatic, autoimmune and cryptogenic. Of note, nearly 60% of patients fell under the recently re-defined umbrella of steatotic liver disease, mirroring the global epidemiological shift from viral to metabolic/toxic causes [2, 3].

In previous studies, a weak correlation between MELD score and FLIS was reported in one study, while another demonstrated a strong correlation [15, 17]. In contrast, our study did not reveal any significant correlation. The reason behind this difference between studies may be results to the nature of the MELD score, which was developed to determine the need for liver transplantation in end-stage liver disease. In contrast to that, our study cohort was heterogeneous with different stages of liver diseases, and not all patients needed transplantation. The ease of use of FLIS in a heterogeneous patient group and its ability to provide functional information may allow it to have a wider range of applications than MELD in clinical practice.

**Fig. 5:**
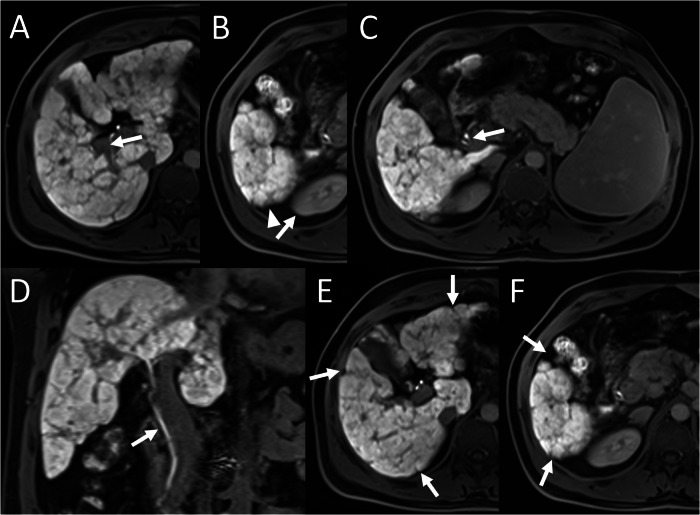
A 37-year-old male patient with chronic hepatitis B underwent abdominal MRI using gadoxetic acid. FLIS was calculated as 6/6 based on: (**A**) portal vein signal intensity (arrow) (score 2), (**B**) liver parenchymal enhancement (arrowhead and arrow) (score 2), and (**C**, **D**) contrast excretion into the extrahepatic bile ducts (arrow) (score 2). Despite the maximum FLIS, FibroScan^®^ indicated advanced fibrosis (F4). **E**, **F** The capsular retraction and heterogeneous parenchyma (arrows) reduced the FLIS-H to 6/8, better reflecting fibrosis severity

**Fig. 6:**
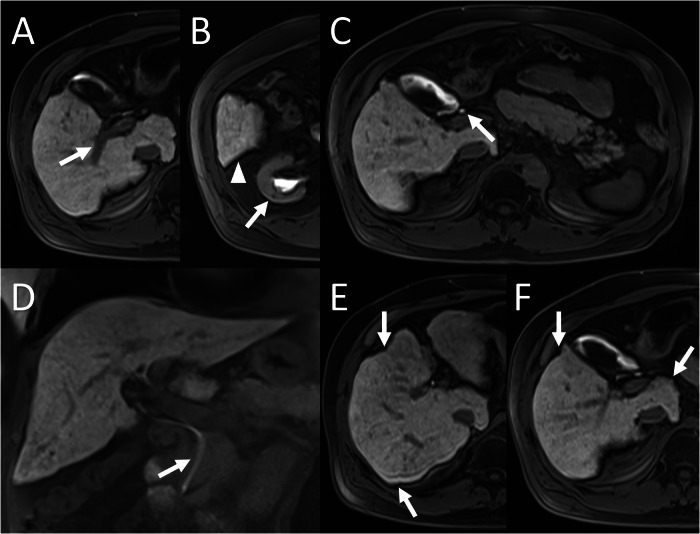
A 64-year-old male patient with ALD, showing maximal FLIS 6/6 despite having F4 fibrosis on FibroScan. **A**–**D** All FLIS components scored 2. **E**, **F** Parenchymal heterogeneity with capsular retraction (arrows) reduced FLIS-H to 6/8, better aligned with the fibrosis stage

A unique contribution of the current study is the incorporation of parenchymal heterogeneity into FLIS. Hepatobiliary-phase heterogeneity has long been recognised as a macro-morphological hallmark of liver diseases; its severity increases in parallel with Child–Pugh class [18]. Yet the original FLIS does not include heterogeneity as a parameter. We observed patients with a maximal FLIS of 6 who nevertheless showed pronounced capsular retraction and coarse inhomogeneity (Figs. 5 and 6). By the integration of two, one or zero points to homogeneous, heterogeneous-without-retraction and heterogeneous-with-retraction patterns into FLIS, FLIS-H (range 0–8) was created. The modified score improved correlation strength with Child–Pugh (ρ = −0.4167 vs −0.2664), ALBI (ρ = −0.5243 vs −0.3076) and fibrosis stage (ρ = −0.5270 vs −0.3207). Steiger’s *z*-statistics confirmed that these gains were statistically significant for fibrosis, Child–Pugh and ALBI (all *p* ≤ 0.05), whereas the modest improvement over MELD did not reach significance. These results emphasise that a rapid visual appraisal of heterogeneity can supply clinically valuable structural information that complements the functional emphasis of the original FLIS.

From a clinical workflow perspective, FLIS-H retains the key advantages that have made FLIS attractive: no additional sequences, no post-processing software and < 30 s of interpretation time. Whereas quantitative T1 mapping, MR elastography or proton-density fat fraction demand specialised sequences and vendor-specific analysis platforms, FLIS-H can be extracted by any radiologist from the standard hepatobiliary phase already acquired for lesion detection. Importantly, this simplicity is paired with excellent reproducibility, as evidenced by the excellent inter-reader agreement observed in our study. The score, therefore, offers an immediate, cost-free avenue for incorporating objective liver-function data into routine reports and multidisciplinary discussions.

The present study has limitations. First, its retrospective, single-centre design and modest sample size limit its generalizability. Second, our study cohort was heterogeneous with suspected liver disease and various etiologies under follow-up. Studies with a more homogeneous patient group may increase the correlation of FLIS and FLIS-H with clinical scores and fibrosis grade. In addition, the fact that the Child–Pugh score includes a subjective parameter such as encephalopathy is a factor that limits the reliability of the correlations obtained with this score, but similar results were indicated with the previous studies. Moreover, liver fibrosis was assessed by transient elastography rather than liver biopsy or MR elastography, which represent reference standards. MRI examinations were also performed on both 1.5-T and 3-T scanners, which may introduce technical variability, but this reflects real-world clinical practice. Although the correlations we obtained were weak to moderate, it should be noted that most correlations reported in the literature have also been moderate. Importantly, the main point of our study is that correlations became statistically more significant with FLIS-H, highlighting its potential clinical utility. Finally, although the median time interval between FLIS evaluation and FibroScan^®^ examination was 4 days, a small subset of examinations approached the upper limit of 90 days. Such intervals may allow progression or fluctuation in liver diseases. Therefore, the correlation between FibroScan^®^ and FLIS performed consecutively may provide more reliable results.

In conclusion, incorporating parenchymal heterogeneity into the FLIS, FLIS-H provides a simple, non-invasive metric that correlates more strongly with elastography-defined fibrosis and clinical prognostic scales. FLIS-H preserves the speed and accessibility of FLIS while potentially broadening its pathophysiological scope. Due to the retrospective design and moderate strength of correlations, further prospective and multi-centre studies are warranted to validate these results and establish the role of FLIS-H in routine clinical assessment.

## Data Availability

The datasets generated and analysed during the current study include sensitive patient information and therefore cannot be made publicly available in their entirety. A partial, de-identified dataset that supports the conclusions of this article may be shared upon reasonable request to the corresponding author, subject to approval from the Institutional Research Ethics Committee.

## Abbreviations

ALBI: Albumin–bilirubin
ALD: Alcohol-related liver disease
CLD: Chronic liver disease
FLIS: Functional liver imaging score
FLIS-H: Functional liver imaging score with parenchymal heterogeneity
Gd-EOB-DTPA: Gadoxetic acid
INR: International normalised ratio
MASLD: Metabolic dysfunction-associated steatotic liver disease
MELD: Model for end-stage liver disease
